# Exploring the link between sedentary behavior and cognitive decline: a comprehensive study combining Mendelian randomization and animal model experiments

**DOI:** 10.3389/fpsyg.2024.1407846

**Published:** 2024-10-14

**Authors:** Yupeng Bai, Mengke Liu, Yan Fang, Ruonan Zhan

**Affiliations:** ^1^Medical Image Center, The Third Affiliated Hospital of Anhui Medical University, Hefei, Anhui, China; ^2^Hubei Key Laboratory of Geriatric Anesthesia and Perioperative Brain Health, Department of Anesthesiology and Pain Medicine, Wuhan Clinical Research Center for Geriatric Anesthesia, Tongji Hospital, Tongji Medical College, Huazhong University of Science and Technology, Wuhan, China; ^3^Department of Ultrasound, Huashan Hospital, Fudan University, Shanghai, China

**Keywords:** cognitive function, time spent watching television, physical exercise, synaptic plasticity, multivariable Mendelian randomization

## Abstract

**Objective:**

The causal link between detrimental behaviors and cognitive performance remains unclear. This research seeks to investigate the causal impact of adjustable lifestyle factors on cognitive deterioration, including frequency of alcohol intake, onset of smoking, and sedentary activities like prolonged television viewing.

**Methods:**

This research combines large-scale genetic data obtained from univariable and multivariable Mendelian randomization analyses with experimental findings obtained from animal models.

**Results:**

Our findings reveal that the odds ratio (OR) for cognitive function deterioration was 0.445 (inverse variance weighted [IVW] 95% CI: 0.370 to 0.536, *p* < 0.001) for each standard deviation increase in television watching time. After adjustment for body mass index (BMI), number of days walked /moderate activity over 10+ min and education in Multivariable Mendelian Randomization (MVMR), only the genetic predisposition to increased television watching time remained significantly associated with worse cognitive function (OR 0.659, 95% CI: 0.452 to 0.960, *p* = 0.030). The other two habits had no significant effects. Sensitivity analyses have confirmed that genetic pleiotropy did not influence the results. To further explore the relationship between sedentary behavior and cognitive function, as well as the underlying mechanisms, we conducted a restricted cage housing experiment and a physical exercise training experiment in mice. The results showed that physical exercise significantly improved spatial memory, as assessed by the Morris water maze, and increased exploratory interest, as evaluated by the open field test (OFT) and the elevated plus-maze test, compared to the sedentary control group. These cognitive advantages may be mediated through mechanisms involving free radical scavenging and enhanced synaptic plasticity.

**Conclusion:**

Our research provides genetic evidence indicating that extended television viewing is linked to an elevated risk of cognitive decline. Additionally, experimental data from mouse models suggest that physical exercise can counteract cognitive decline and anxiety-like behaviors induced by sedentary behavior. This protective effect is likely mediated by reactive oxygen species (ROS)-dependent mechanisms that enhance synaptic plasticity within the hippocampus.

## 1 Introduction

The increasing proportion of elderly people in the population has made cognitive decline a significant public concern. Cognitive health plays a vital role in preserving the quality of life for older adults. It allows them to participate in social interactions, maintain independence, recover more rapidly from illnesses or injuries, and adjust to the natural decline in organ function (Quinn et al., 2020; Clare et al., 2017).

Therefore, interventions that could even modestly delay the onset of cognitive function decline would have a major impact on public health.

Current epidemiological and experimental data suggest that modifiable lifestyle factors may play pivotal roles in brain health, potentially preventing or decelerating cognitive decline and the progression of dementia (Anstey et al., 2013; Song et al., 2022). Among these, smoking (Peters et al., 2008), drinking alcohol (Peters et al., 2008), and prevalent recreational activities such as watching television (TV; Gao et al., 2021; Patterson et al., 2018; Thorp et al., 2011; Wijndaele et al., 2017) have been extensively studied due to their ubiquity. However, the causal connections between these activities and cognitive function have not been conclusively established. Moreover, it remains uncertain which among these lifestyle factors predominantly impact cognitive health adversely.

Mendelian randomization (MR; Burgess and Labrecque, 2018), which employs single nucleotide polymorphisms (SNPs) as instrumental variables to evaluate the effects of exposures on specific outcomes, provides a cost-efficient and ethically sound alternative to randomized controlled trials for investigating the influence of smoking and alcohol consumption on cognitive function. Multivariable Mendelian Randomization (MVMR; Zhang et al., 2021), which combines multiple SNPs into a single model, extends the traditional MR approach. It allows for the exploration of potential causal effects of various exposures on an outcome and determines which of these factors are primarily responsible for these effects.

In industrialized countries, the most common sedentary behavior is watching TV. Passive sedentary behavior, such as watching television, is associated with a higher risk of depression, unlike sedentary behavior involving mentally engaging activities, such as playing computer (Grontved and Hu, 2011; Hu et al., 2003). The purpose of this study is to investigate the impact of one representative activity within the broader category of sedentary behaviors—watching TV—on cognitive function.

Animal experiments permit an in-depth exploration of how sedentary behavior affects cognitive function through specific biological pathways in a controlled environment.

In this study, we used MR analysis to determine which lifestyle factors are the most strongly linked to impaired cognitive function. Our findings have identified extended television viewing as a significant risk factor for cognitive decline, likely due to its association with both low energy expenditure and diminished cognitive stimulation. To investigate the underlying mechanisms, we constructed an animal model to examine whether low physical activity affects the behavioral performance of mice. Subsequently, treadmill training was administered to determine if this intervention could reverse the observed effects.

## 2 Materials and methods

### 2.1 Study design

**Figure 1:**
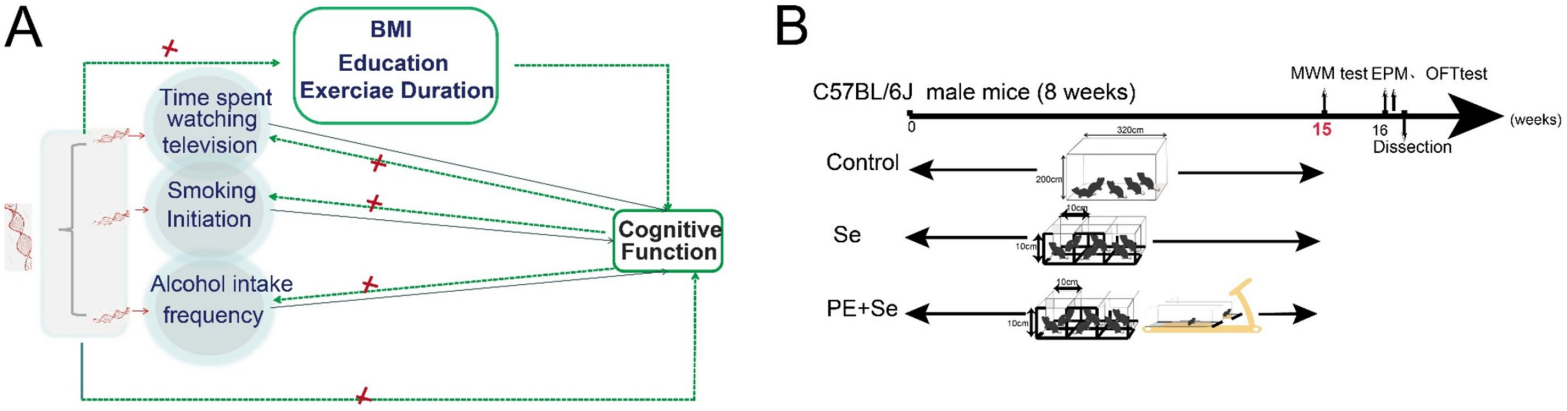
**(A)** Overview and assumptions of the Mendelian randomization study design. **(B)** Animal experiment workflow diagram.

All two-sample MR analyses in this study follow the STROBE-MR guidance (Skrivankova et al., 2021). Figure 1A shows an overview of our study design. It is important to note that our analyses were not pre-registered, which means that the findings should be interpreted as exploratory in nature.

We initially determined whether the three typical lifestyle factors have a causal effect on cognitive function using univariable Mendelian and MVMR methods. Then, potential for reverse causality was investigated to examine whether genetically proxied cognitive function had a causal effect on each of the three typical categories of lifestyle factors. Additionally, we searched in the PhenoScanner database to identify previously reported associations between the instrument SNPs and potential confounders. When the search revealed genome-wide significant associations (*p* < 5 × 10^−8^) with shared risk factors for smoking, alcohol consumption, television watching, and cognitive function, we applied MVMR to satisfy the second assumption of MR analysis. Finally, MVMR adjusted for the potential confounders screened by PhenoScanner database search were performed to disentangle which one of the three lifestyle factors exerted independent and predominant effects on cognitive function.

To investigate the underlying mechanisms, we constructed a low physical activity model mouse to examine whether treadmill training improves cognition. There are several methods to model sedentary behavior. The Physical Inactivity (PI) restriction cage is the most widely used method. This is primarily due to its lower susceptibility to external influencing factors, such as stress from social isolation, dietary effects, or impaired motor function. The design for the animal experiment is illustrated in Figure 1B.

### 2.2 Data sources

We extracted SNPs that exhibited strong associations with the three target phenotypes–smoking initiation, time spent watching television, and frequency of alcohol consumption–from genome-wide association studies (GWASs) conducted by the GWAS and Sequencing Consortium of Alcohol and Nicotine use (GSCAN) and MRC-IEU Consortium. When selecting the SNPs and their summary statistics, we followed the principle that the study only included individuals of European ancestry, and the study with the same characteristics had the largest sample size.

We obtained summary-level data on the outcome (cognitive function) from a GWAS in the within-family GWAS consortium Project, comprising data of 6,719,661 SNPs from 22,593 participants of European ancestry. The summary statistics for each phenotype of interest were obtained from GWASs, as detailed in Table 1.

**Table 1:** Details of studies included in this MR study.

Traits	Consortium	Cases	Control	Sample size	Year	Population	Pubmed ID	Web source
Cognitive function	within-family GWAS consortium	NA	NA	22,593	2022	European	NA	https://gwas.mrcieu.ac.uk/datasets/ieu-b-4838/
BMI (SD, ~4.8 kg/m^2^)	GIANT	NA	NA	681,275	2018	European	30,124,842	https://gwas.mrcieu.ac.uk/datasets/ieu-b-40/
Smoking initiation	GSCAN	311,629	321,173	607,291	2019	European	30,643,251	https://gwas.mrcieu.ac.uk/datasets/ieu-b-4877/
Time spent watching television (TV)	MRC-IEU	NA	NA	437,887	2018	European	NA	https://gwas.mrcieu.ac.uk/datasets/ukb-b-5192/
Alcohol intake frequency	MRC-IEU	NA	NA	462,346	2018	European	NA	https://gwas.mrcieu.ac.uk/datasets/ukb-b-5779/
Qualifications: College or University degree	MRC-IEU	148,722	309,357	458,079	2018	European	NA	https://gwas.mrcieu.ac.uk/datasets/ukb-b-16489/
Number of days/week of moderate physical activity 10+ minutes	MRC-IEU	NA	NA	440,266	2018	European	NA	https://gwas.mrcieu.ac.uk/datasets/ukb-b-4710/
Number of days/week walked 10+ minutes	MRC-IEU	NA	NA	454,783	2018	European	NA	https://gwas.mrcieu.ac.uk/datasets/ukb-b-4886/

GWAS, genome-wide association study; GSCAN, GWAS and Sequencing Consortium of Alcohol and Nicotine use; MR, Mendelian randomization; MRC-IEU, The Medical Research Council Integrative Epidemiology Unit at the University of Bristol; SNP, single-nucleotide polymorphism; BMI, body mass index; GIANT, Genetic Investigation of Anthropometric Traits; SD, standard deviation.

### 2.3 Instrumental variable selection

Independent SNPs linked to each exposure of interest at genome-wide significance (*p* < 5 × 10^−8^) were selected as genetic instruments from the GWAS datasets (Jiang et al., 2023). After that, SNPs in linkage disequilibrium (LD, which is defined as r^2^ ≥ 0.001 or within 10,000 kb) were clumped to obtain independent SNPs (Shen et al., 2021). We then extracted the SNPs from the summary statistics of the outcome, cognitive function. Additionally, data harmonization was performed to align the SNP alleles between the exposure and outcome.

The F statistic was used to evaluate the weak instrumental variable bias, and when *F* ≤ 10 indicates weak instrumental variables, the corresponding SNPs were deleted (Burgess et al., 2011). The variance (R^2^) in each exposure explained by SNPs was calculated through the formula as follows: [2 × EAF × (1−EAF) × *β*^2^]/ [2 × EAF × (1−EAF) × (β^2^) + 2 × EAF × (1−EAF) × N × SE (β)^2^] (Wu et al., 2020). F statistic calculation formula is as follows: F = [(n−K−1) / K] × [R^2^ / (1−R^2^)] (Burgess et al., 2011), where EAF denotes the effective allele frequency, β denotes the coefficient indicating effect size, SD denotes to the standard deviation within the sample size of the exposure dataset, K denotes the number of SNPs, and R^2^ is the proportion of variance in the exposure dataset explained by the SNPs.

After the above steps were completed, the remaining SNPs were selected as the genetic instruments for the following UVMR and MVMR analyses. We also conducted reverse-directional MR analyses to investigate the potential causal impact of genetically proxied cognitive function on the three lifestyle factors.

### 2.4 UVMR analysis

In this study, the inverse variance weighted (IVW) method, MR-Egger regression, and weighted median estimator (WME) were used for Mendelian randomization analysis (Marouli et al., 2019). The IVW method employs a meta-analysis approach to aggregate the Wald ratios of the causal effects of each SNP, based on the assumption that all SNPs are valid instrumental variables without directional pleiotropy (Cui et al., 2020). We used the IVW method as the primary analysis to combine the effects (i.e., Wald ratios) of individual SNPs, while the other two methods were employed to assess the robustness of the main results (Ong and MacGregor, 2019). If the results from all three analytical methods are generally consistent, conclusions regarding causality can be drawn with increased confidence and persuasiveness (Zhang et al., 2023).

As we tested three exposures, we applied a Bonferroni-corrected threshold of *p* < 0.05/3 = 0.017 to indicate significant associations in the primary analysis (Magnus et al., 2020). In the univariable MR analysis, a *p*-value below the Bonferroni-corrected threshold was considered statistically significant, whereas *p*-values exceeding the Bonferroni-corrected threshold but below 0.05 were considered suggestive of statistical significance (Wu et al., 2020).

### 2.5 Heterogeneity, pleiotropy, and sensitivity analysis

During the MR analysis, we conducted heterogeneity, pleiotropy, and sensitivity analyses to ensure the robustness of the MR estimates. Cochran’s Q test was performed to evaluate heterogeneity (Greco et al., 2015), with a p-value greater than 0.05 suggesting no evidence of heterogeneity.

Additionally, the MR-Pleiotropy Residual Sum and Outlier (MR-PRESSO) method was applied (Verbanck et al., 2018), and a leave-one-out analysis was conducted to identify outliers that might affect the MR estimates (Burgess and Thompson, 2017). An intercept analysis using MR-Egger regression was performed, where a p-value less than 0.05 indicated the presence of pleiotropy. Following this, the Mendelian Randomization Pleiotropy RESidual Sum and Outlier (MR-PRESSO) method was employed to adjust for outliers by removing one or more pleiotropic SNPs and rerunning the MR analyses to provide refined estimates of causal associations.

### 2.6 Complementary analysis

To determine whether cognitive function had a causal impact on our proposed exposures, we also performed MR analysis in the opposite direction. Following the same method outlined previously, a distinct set of SNPs was selected as genetic instruments for cognitive function. Only two SNPs related to cognitive function were identified as genetic variants. To evaluate pleiotropy analysis and detect the direction of MR-Egger regression and WME, we set a genome-wide significance level of *p* < 5 × 10^−7^ level to extract SNPs from the cognitive function GWAS datasets.

### 2.7 MVMR analysis

MVMR analyses were conducted to determine which of these lifestyle factors primarily contributed to the causal associations with cognitive function (Figure 1A).

First, we combined SNPs into an integrated proxy for the three exposures. Subsequently, MVMR analysis was carried out to account for indirect pathways that might lead to correlated pleiotropy. A search of the PhenoScanner database identified potential confounders that have genome-wide significant associations (*p* < 5 × 10^−8^) with smoking, alcohol consumption, television watching, and cognitive function. The search revealed associations between the instruments and education and obesity-related traits. Indeed, numerous studies have demonstrated that physical exercise exerts a protective impact on cognitive function (Brasure et al., 2018; Iso-Markku et al., 2024).

Therefore, we selected SNPs for education from the MRC-IEU dataset (458,079 participants), SNPs for BMI from the GIANT Consortium (681,275 participants), and SNPs for number of days walked/moderate activity (10+ min) from the MRC-IEU dataset (454,783 participants) to perform the MVMR analyses (Table 1).

### 2.8 Animals

All animal experiments were performed according to the guidelines set by the National Ministry of Health and were approved by the Laboratory Animal Center of Shanghai Tenth People’s Hospital (SHDSYY-2023-3429-82278). Eight-week-old male C57BL/6 J mice were purchased from SLNAC Experimental Animal Technology Co., Ltd., Shanghai, China.

To simulate sedentary behavior, we used a PI cage, as designed by Kim et al. (2022), which restricts the mice’s movement.

Mice were randomly divided into three groups: control, sedentary group (Se group), and sedentary+ Physical Exercise group (Se + PE). The mice in the control group were housed in standard cages (320 × 200 × 200 cm) with a maximum of five mice per cage. The sedentary group mice were kept in PI cages (10 × 10 × 10 cm) for 15 weeks. Mice in the Se + PE group were also housed in PI cages but underwent treadmill exercise throughout the 15-week period (Figure 1B). The treadmill exercise program was conducted 4 days per week, with each session lasting 40 min, at a speed of 10 meters per minute. The modeling experiment lasted for approximately 15 weeks. In the 16th week, the Morris water maze (MWM) test was conducted (5 days of training followed by a hidden platform test on the sixth day), followed by the Elevated Plus Maze (EPM), and the Open Field Test (OFT). After the behavioral tests, the mice were subjected to cardiac perfusion, and their brains were collected for histological analysis.

#### 2.8.1 Behavioral tests

Sedentary behavior can affect cognitive function not only through physiological mechanisms, such as metabolic dysfunction but also by exacerbating cognitive decline through behavioral and psychological pathways, such as increased anxiety and depression (Zhai et al., 2015). The anxiety state of mice may directly affect their performance in cognitive tests. We use three classic behavioral experiments—the Morris water maze, the EPM, and the open field test—to evaluate the behavioral changes in mice from multiple dimensions, including spatial memory, anxiety, and exploratory behavior.

The EPM test was executed following the methodology described by Komada et al. (2008), with 5 min. The number of entries and the time spent in each arm were measured using Any-Maze software (Stoelting Co., Wood Dale, IL, USA).

The MWM was conducted as per the protocol established by Frame et al. (2019), utilizing a pool measuring 120 cm in diameter and 50 cm in height, accompanied by a submerged platform. Spatial learning and memory were assessed through training trials, conducted four times daily over 4 days, followed by a hidden platform test lasting 60 s.

According to the method of Ma et al. (2023), the OFT was examined in a box (50 cm × 50 cm × 50 cm). The immobility time and the duration spent in the center zone were automatically recorded by Any-Maze software.

#### 2.8.2 Western blotting

Briefly, a total of 20 μg of protein extracts from the hippocampus of mice were subjected to separation by 12% sodium dodecyl sulfate polyacrylamide gel electrophoresis (SDS-PAGE) and then transferred onto a 0.45 μm Polyvinylidene fluoride (PVDF) membrane (Millipore, MA, United States). Western blot analysis was carried out and analyzed according to previously established protocols. The antibodies were as follows: synaptophysin (Cell Signaling Technology, Danvers, MA, United States) and rabbit PSD95 antibodies (Cell Signaling Technology, Danvers, MA, United States), and *β*-tubulin (ABclonal, Wuhan, China). The membrane was incubated for a duration of 1 h at room temperature with HRP-conjugated anti-rabbit and anti-mouse IgG (1:10000, ABclonal), followed by washing. The bands were subsequently observed using electrochemiluminescence (ECL) detection reagents (ABclonal, Wuhan, China) on a chemiluminescence instrument (Image Quant LAS 4000, GE Healthcare, Pittsburgh, PA, United States). The intensity of the bands was analyzed using ImageJ software.

#### 2.8.3 DHE staining

To assess intracellular superoxide levels in the hippocampal region, we utilized *in situ* dihydroethidium (DHE) fluorescence staining (1 μM, Invitrogen-Molecular Probes, Eugene). Tissue sections were incubated with DHE diluted in phosphate-buffered saline (PBS) for 30 min at room temperature in darkness. Density calculations of the images were performed using ImageJ software.

### 2.9 Statistical analysis

All statistical analyses were performed using R software (v3.6.3) with the “TwoSampleMR” (v0.5.6), “MendelianRandomization” (v0.5.1), and “MRPRESSO” (v1.0) packages.

Data are presented as means ± SEM. GraphPad Prism version 6.0 was used to perform all of the statistical analyses. The results from the Western blot and fluorescent staining were tested using repeated measures of one-way analysis of variance (ANOVA), followed by a Bonferroni *post hoc* test to evaluate the difference between each group. The behavioral results were evaluated statistically using a one-way ANOVA with Tukey’s test corrected for multiple comparisons.

## 3 Results

### 3.1 Eligible SNPs

When extracting SNPs from cognitive function datasets, it was observed that there were 0, 2, and 2 SNPs absent, respectively, accounting for 0, 2, and 1.8% proportions for smoking initiation, alcohol intake frequency, and time spent watching television. After excluding the IVs with linkage disequilibrium, and palindromic SNPs, a total of 73, 88, and 100 SNPs were identified as IVs for smoking initiation, alcohol intake frequency, and time spent watching television, respectively. These SNPs explained 0.48, 1.03, and 0.94% of the phenotypical variance, respectively, and had a sum of F-statistic of 3066.39, 4777.23, and 4152.80, respectively (see Table 2; Supplementary Tables 1–3 for the basic information about SNPs).

**Table 2:** UVMR analysis for genetically causal associations of the three living habits with cognitive function.

Exposure	nSNP	R^2^(%)	F-statistic	MR method	OR	95% CI		*p*
Alcohol intake frequency	88	1.03	4777.22	Weighted Median	0.853	0.726	1.001	0.052
				MR Egger	0.963	0.735	1.261	0.782
				Inverse variance weighted	0.786	0.709	0.870	0.000
Smoking initiation	73	0.48	3066.39	Weighted Median	0.740	0.636	0.861	0.000
				MR Egger	0.607	0.302	1.219	0.165
				Inverse variance weighted	0.793	0.693	0.908	0.001
Time spent watching television	100	0.94	4152.80	Weighted Median	0.401	0.311	0.518	0.000
				MR Egger	0.269	0.112	0.646	0.004
				Inverse variance weighted	0.445	0.370	0.536	0.000

R^2^ represents the total proportion of phenotypic variation that is explained by SNPs in MR analyses. The F-statistic reflects the overall strength of the SNPs as instruments for each socioeconomic trait, with an F-statistic greater than 10 suggesting strong instrumental variables capable of explaining phenotypic variation. CI, confidence interval; IVW, inverse-variance weighted; MR, Mendelian randomization; OR, odds ratio; nSNP, number of single-nucleotide polymorphism; WM, weighted median.

### 3.2 Effect estimations based on IVW UVMR

As shown in Table 2, genetic susceptibility to smoking initiation was positively associated with worse cognitive function (OR 0.793, 95% CI: 0.693 to 0.908, *p* < 0.001). The combined OR for cognitive function was 0.786 (IVW 95% CI: 0.709 to 0.870, *p* < 0.001) and 0.445 (IVW 95% CI: 0.370 to 0.536, *p* < 0.001) for an increase of one standard deviation in alcohol intake frequency and time spent in watching television, respectively. The results regarding the causal associations between the three lifestyle factors and cognitive function as determined by UVMR analyses utilizing three different MR methods are shown in Table 2. The direction of the three methods is consistent. The positive results also survived the Bonferroni correction.

Also, the potential for reverse causality was investigated. We conducted reverse-directional MR analyses to investigate the potential causal effect of genetically proxied cognitive function on each of the three exposures, respectively. SNPs linked to cognitive function were obtained from GWAS datasets at a genome-wide significance threshold (*p* < 5 × 10^−7^). We then discarded the SNPs surrogating for confounding traits with a threshold of r^2^ > 0.80 (confounder-related SNPs detailed in Supplementary Table 4) and retained six SNPs serving as IVs for cognitive function.

Our reverse directional UVMR analyses show that genetically proxied cognitive function has a causal association with none of the lifestyle factors (“smoking initiation”: OR 0.964, 95% CI: 0.909 to 1.022, *p* = 0.214; “alcohol intake frequency”: beta −0.056, 95% CI: −0.136 to 0.024, *p* = 0.167; and “time spent watching television”: beta −0.033, 95% CI: −0.068 to 0.003, *p* = 0.070) based on IVW UVMR model (Supplementary Table 5).

The above result expels the possibility of reverse causality between genetically proxied cognition and any of the three lifestyle factors.

### 3.3 Sensitivity, heterogeneity, and pleiotropy

Table 3 shows that there exists significant heterogeneity regarding the effect estimates of “smoking initiation” (Q = 160.52, I^2^ = 55.14%, *p* < 0.001) and “Alcohol intake frequency” (Q = 112.51, I^2^ = 22.67%, *p* = 0.034) on cognitive function in UVMR analyses. The leave-one-out permutation analysis showed that there was no palpable sensitive SNP driving the estimates (Supplementary Figures 1–3).

**Table 3:** Heterogeneity and pleiotropy for genetically causal associations of the three living habits with cognitive function.

Exposure	nSNP	Heterogeneity			Pleiotropy		
		Q	I^2^(%)	*p*	Intercept	SE	*p*
Alcohol intake frequency	88	112.51	22.67	0.034	−0.0050	0.003	0.115
PPRESSO adjustment	87	102.51	16.1	0.108	0.0023	0.005	0.6044
Smoking initiation	73	160.52	55.14	0.000	0.0070	0.009	0.446
PPRESSO adjustment	71	70.00	49.13	0.000	0.0025	0.009	0.773
Time spent watching television	100	110.52	10.43	0.200	0.0059	0.005	0.253

SE, standard error; nSNP, number of single-nucleotide polymorphism; PPRESSO, pleiotropy residual sum and outlier.

As shown in Table 3, there is no obvious pleiotropy concerning the effect estimates of all the three exposures on cognitive function in UVMR (“smoking initiation”: Egger intercept = 0.007, SE = 0.009, *p* = 0.446; “alcohol intake frequency”: Egger intercept = −0.005, SE = 0.003, *p* = 0.115; and “time spent watching television”: Egger intercept = 0.0059, SE = 0.005, *p* = 0.253). The term pleiotropy refers to the fact that genetic variants can influence multiple traits through different causal pathways. MR has a “no distinct pleiotropy” assumption, which implies that the effects of the genetic variants on the outcome solely come through the effects of genetic variants on the exposure, with great possibility. However, we still make MVMR adjustments for possible confounding factors to enhance our result’s robustness.

As shown in Tables 2, 3, we conducted MR-PRESSO analyses to identify pleiotropic outlying SNPs and determine outlier-adjusted estimates after removing these outlying SNPs (“smoking initiation”: PRESSO-adjusted OR 0.797, 95% CI: 0.751 to 0.888, *p* < 0.001; and “alcohol intake frequency”: PRESSO-adjusted OR 0.757, 95% CI: 0.686 to 0.834, *p* < 0.001). Remarkably, after PRESSO adjustments, we did not observe significant heterogeneity in the results for significant effects of alcohol intake frequency (Q = 102.51, I^2^ = 16.1%, *p* = 0.108). However, there remained significant heterogeneity in the results of smoking initiation and cognitive function following PRESSO adjustments (Q = 70, I^2^ = 49.13%, *p* < 0.001).

**Figure 2:**
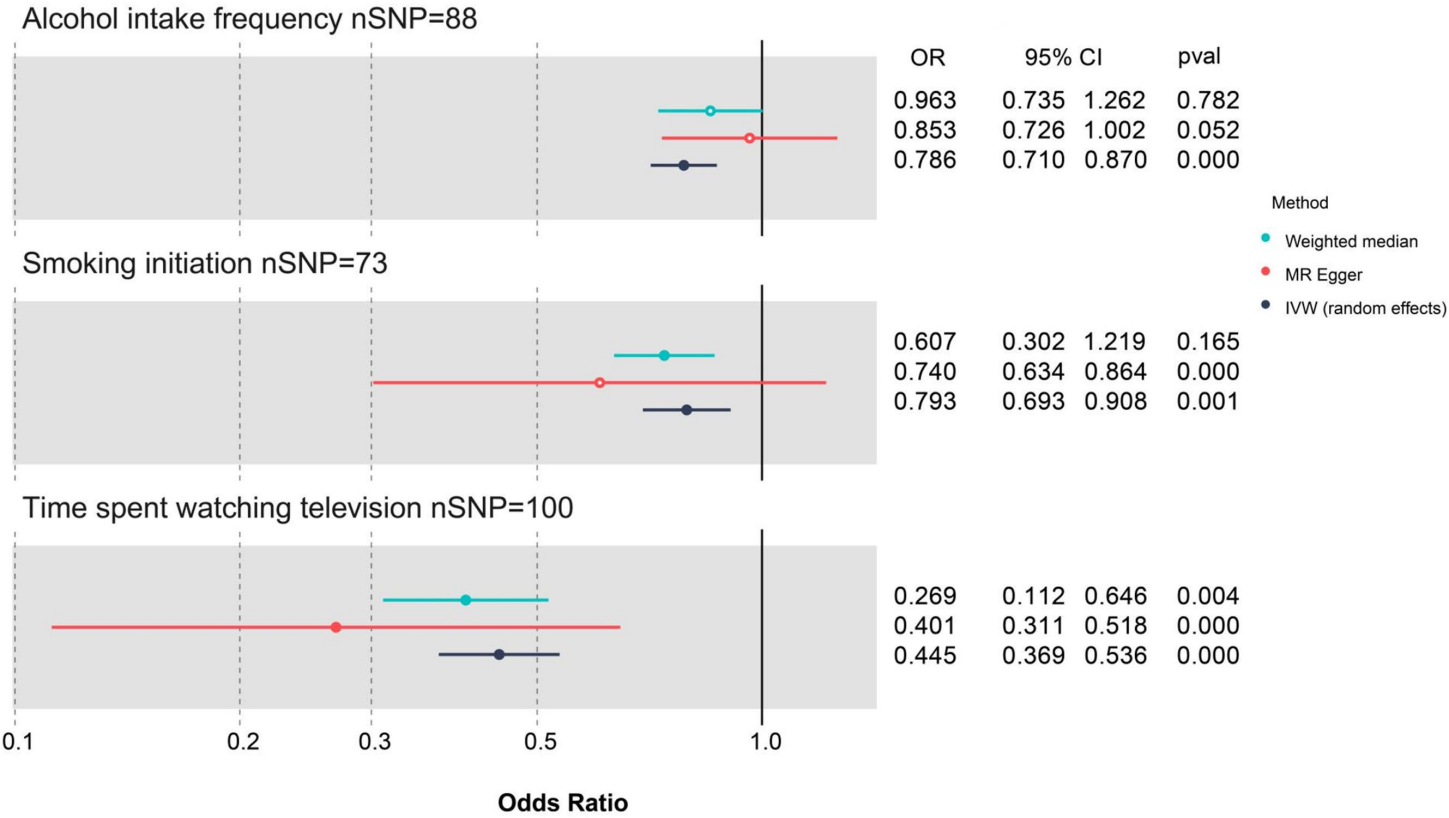
Forest plot for the three lifestyle factors with cognitive function based on three UVMR methods. CI, confidence interval; MR, Mendelian randomization; nSNP, number of single-nucleotide polymorphism; OR, odds ratio; IVW, inverse-variance weighted; UVMR, univariate Mendelian randomization; WM, weighted median. Blue represents the WM method, red represents MR-Egger, and dark blue represents the IVW method.

The results of the effects (*β* values) of the three exposures on cognitive function in UVMR analyses are visualized in three scatter plots (Supplementary Figures 4–6). Moreover, the results of effect estimates for individual SNPs in UVMR analyses are illustrated in three forest plots (Supplementary Figures 7–9), and that for all SNPs is demonstrated in a summarized forest plot (Figure 2).

### 3.4 MVMR results

**Figure 3:**
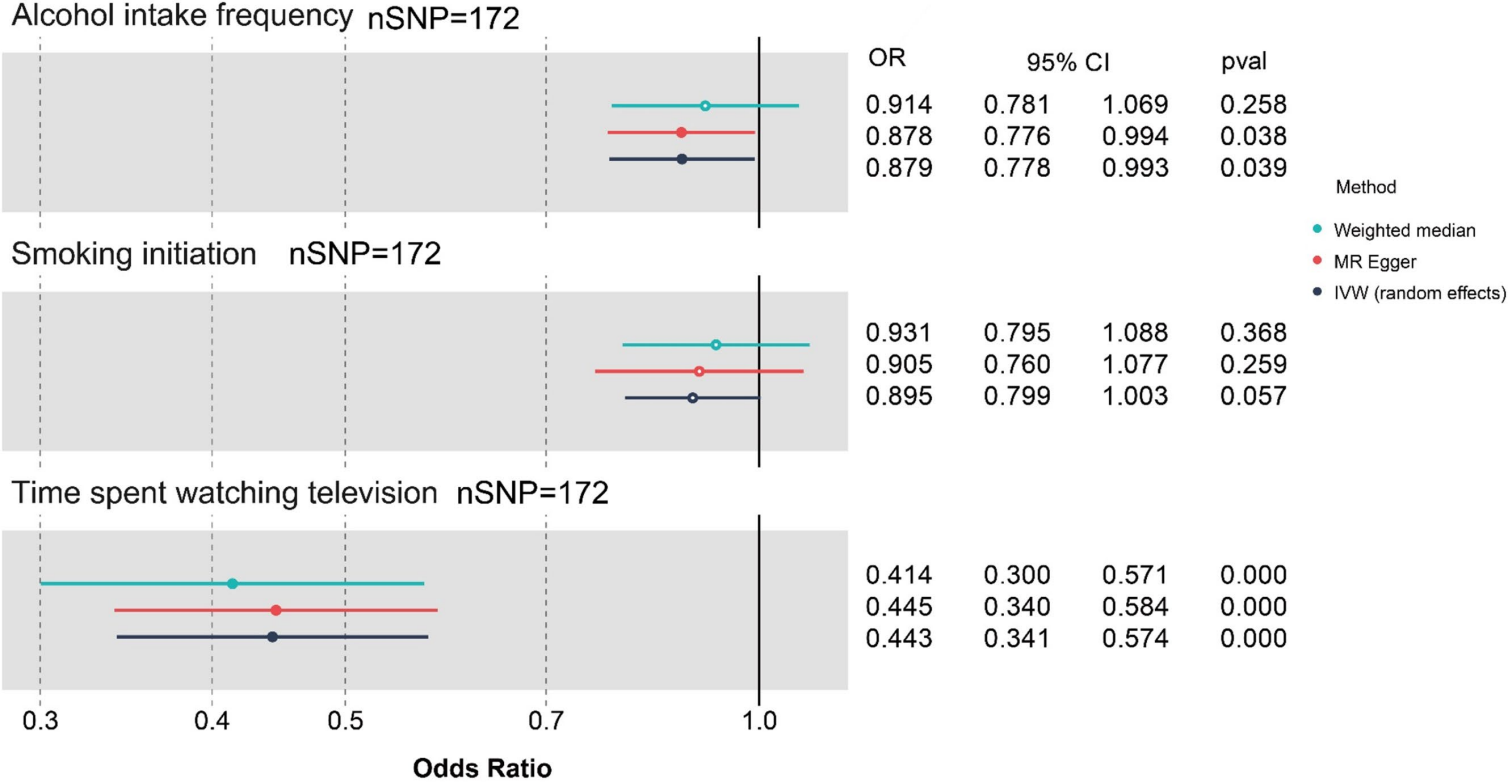
Forest plot for causal associations of the three lifestyle factors with cognitive function based on MVMR. nSNP, number of single-nucleotide polymorphism; OR, odds ratio; CI, confidence interval; IVW, inverse-variance weighted; MR, Mendelian randomization; MVMR, Multivariable Mendelian randomization; Blue represents WM method, red represents MR-Egger, and dark blue represents IVW method.

The results of MVMR analyses based on three exposures are shown in Figure 3 and Table 4. As shown in the figure and table, the more time spent on TV (OR 0.443, 95% CI: 0.341 to 0.574, *p* < 0.001) and the higher the alcohol intake frequency (OR 0.879, 95% CI: 0.778 to 0.993, *p* = 0.039), the greater the negative effects are on cognitive function after adjusting for “smoking initiation” (OR 0.894, 95% CI 0.798 to1.003, *p* = 0.057).

**Table 4:** Causal effects of the three living habits on cognitive function based on IVW MVMR.

Exposure	nSNP	β	SE	*p*	OR	95%CI UP	95%CI Down
MVMR for the three habits							
Alcohol intake frequency	172	−0.129	0.062	0.039	0.879	0.993	0.778
smoking initiation	172	−0.111	0.058	0.057	0.895	1.003	0.798
Time spent watching television	172	−0.815	0.133	0.000	0.443	0.574	0.341
Adjustment for BMI and education							
Alcohol intake frequency	431	−0.101	0.080	0.205	0.904	0.773	1.057
Smoking initiation	431	0.070	0.066	0.290	1.073	0.942	1.221
Time spent watching television	431	−0.417	0.192	0.030	0.659	0.452	0.960
BMI	431	0.010	0.045	0.831	1.010	0.925	1.103
Number of days/week of moderate physical activity 10+ minutes	431	−0.069	0.067	0.299	0.933	0.818	1.064
Qualifications: College or University degree	431	1.187	0.245	0.000	3.277	2.027	5.297

IVW, inverse-variance weighted; MVMR, Multivariable Mendelian randomization; SE, standard error; OR, odds ratio; CI, confidence interval; nSNP, number of single-nucleotide polymorphism; BMI, body mass index.

**Figure 4:**
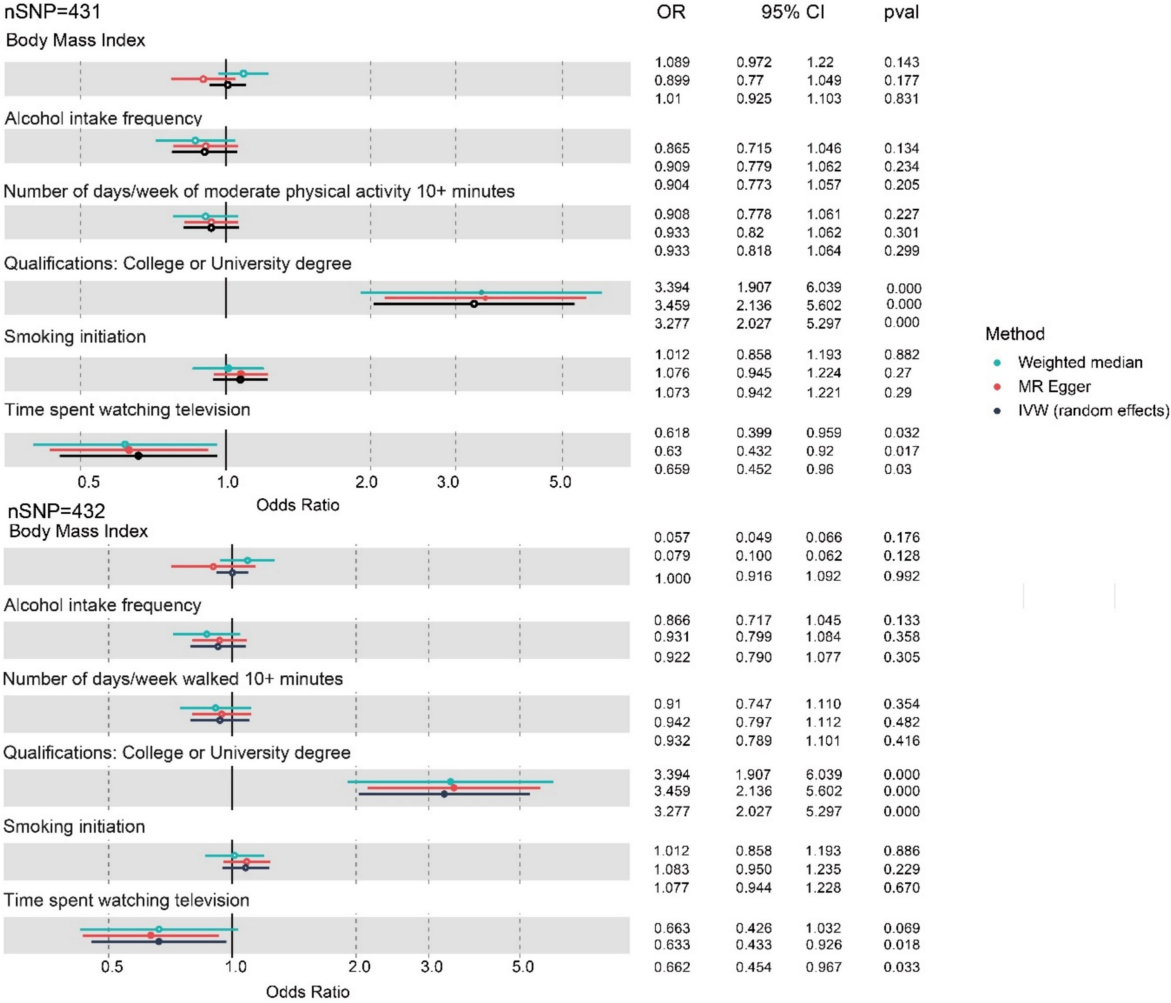
Forest plot for the three lifestyle factors with cognitive function adjusted for BMI, number of days/weeks of physical exercise, and education based on MVMR. nSNP, number of single-nucleotide polymorphism; OR, odds ratio; CI, confidence interval; IVW, inverse-variance weighted; MR, Mendelian randomization; MVMR, multivariable Mendelian randomization. Blue represents the WM method, red represents MR-Egger, and black represents the IVW method.

Further, the PhenoScanner search detected associations of IVs (instrumental variables) of the three exposures with education and obesity-related traits. Numerous studies have demonstrated that physical exercise exerts a protective effect on cognitive function (Brasure et al., 2018; Iso-Markku et al., 2024). Then, multivariable MR adjusted for the education, BMI, and physical exercise duration was performed to estimate the causal association between the three lifestyle factors and worse cognitive function further. The genetic liabilities for time spent watching television (OR 0.659, 95% CI: 0.452 to 0.960, *p* = 0.03) showed a positive association with decreased cognitive function after adjusting for potentially related pleiotropy by education and BMI in MVMR analysis (Figure 4; Table 4). It appears that this factor plays a predominant role in the associations of the three typical lifestyle factors with diminished cognitive function.

However, after adjusting for BMI, education, and exercise jointly, the association between alcohol intake frequency (OR 0.904, 95% CI: 0.773 to 1.057, *p* = 0.205) and the risk of worse cognitive function was not significant.

Spending more time watching TV is associated with a one-third higher risk of future cognitive decline compared to frequent alcohol consumption, regardless of smoking habits. Our study suggests a causal potential association between time spent watching television and worse cognitive function. It is evident from our results that education has a protective effect on cognition, which is also consistent with the majority of the literature.

### 3.5 Behavioral test

**Figure 5:**
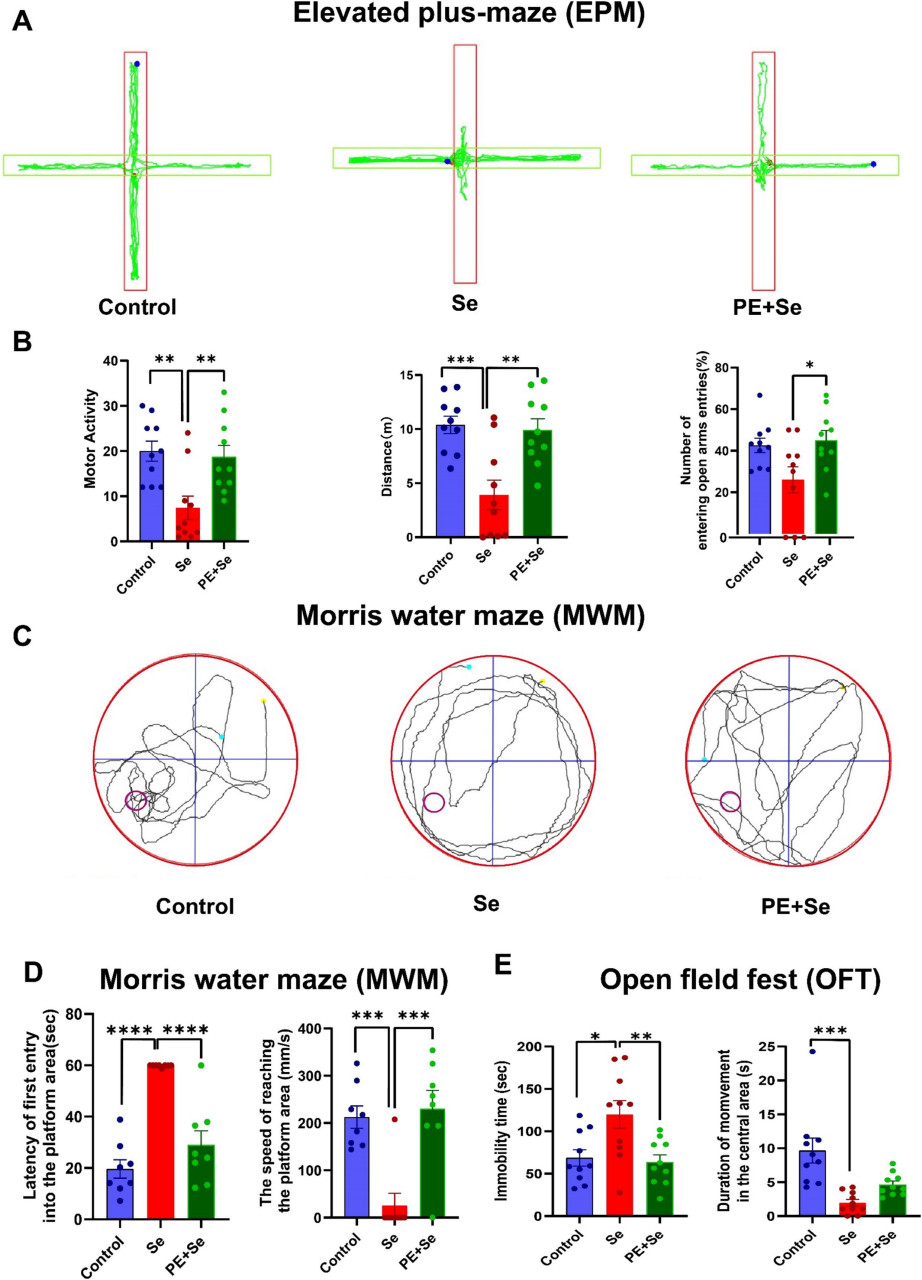
Behavioral performance in different groups (Control, Se, PE + Se). (A) Representative trace plots of EPM. (B) Motor activity, total distance, Proportion of the number of entering the open arm in the EPM. (C) Representative trace plots of MWM. (D) Latency of the first entry into the platform and the mean speed of reaching the platform area. (E) Immobility time and duration of movement in the central area of OFT. All data are presented as the mean ± SEM (Control mice, *n* = 10; Se mice, *n* = 10; PE + Se mice, *n* = 10). Data were analyzed using one-way ANOVA with Tukey’s post-hoc test. *p < 0.05, **p < 0.01, ***p < 0.001, ****p < 0.0001.

We conducted three behavioral tests to assess cognitive function and exploratory behavior in mice. First, in EPM, we observed that motor activity, total distance traveled, and the number of entries into open arms were significantly decreased in the sedentary mice (Se) compared to both the control mice (Control) and the sedentary mice subjected to physical exercise (Se + PE) (Figures 5A,B). Second, MWM results, aimed at evaluating spatial memory, revealed that the Se group had longer latencies before the first entry onto the platform and lower average speeds in reaching the platform area (Figures 5C,D). Third, in the OFT designed to assess exploratory behavior, the immobility time was significantly higher in the Se group as compared to the Control and Se + PE mice. However, the time spent in the central area was not significantly different between the Se and Se + PE groups, suggesting a diminished exploratory interest in the Se group (Figure 5E).

### 3.6 Histological tests

**Figure 6:**
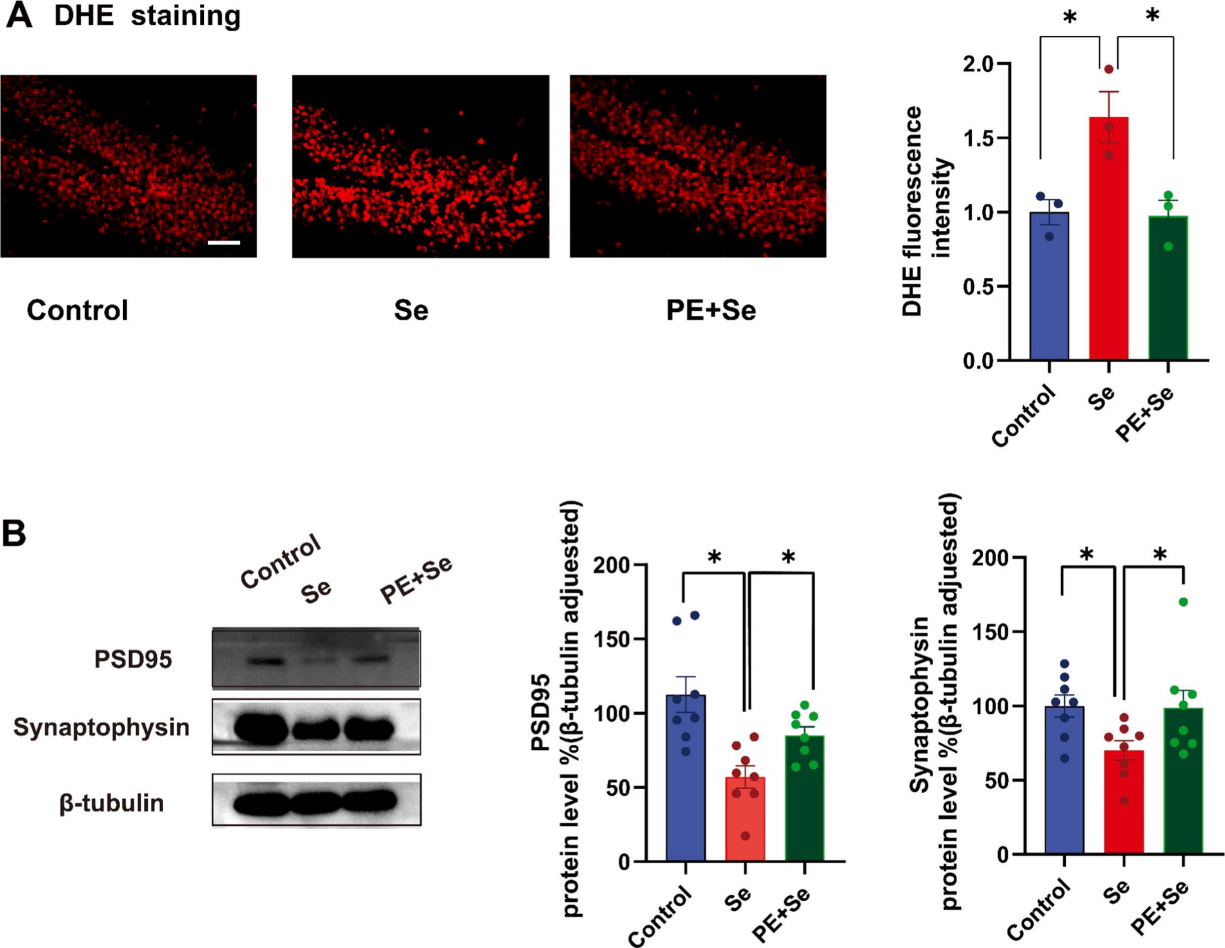
DHE staining and the levels of key proteins reflecting synaptic structure and function, Syn and PSD95 (Control, Se, PE + Se). (A) Hippocampal DHE staining and quantitative analysis. (B) Representative blots and blot density of synaptophysin and PSD95 in the hippocampus. All data are presented as the mean ± SEM (Control mice, *n* = 8; Se mice, *n* = 8; PE + Se mice, *n* = 8). Bar = 100 μm. Data were analyzed by repeated measures of one-way analysis of variance (ANOVA), followed by the Bonferroni *post-hoc* test. *p < 0.05.

DHE staining results indicated a significant elevation in hippocampal superoxide levels in the Se group in comparison to both the control and Se + PE groups (Figure 6A). Additionally, Western blot analysis of synaptophysin and PSD95 protein levels in the hippocampus showed a significant upregulation in the Se + PE group relative to the Se group, indicating enhanced synaptic integrity (Figure 6B).

## 4 Discussion

With the aging of the global population, it is expected that the number of dementia cases will increase in the coming decades. To date, there are no effective pharmacological therapies for treating age-related cognitive impairment, underscoring the critical importance of preventive strategies. The relationships between diet, physical activity, and other lifestyle factors and cognitive function have been extensively studied, with growing evidence supporting the role of these determinants in cognitive decline (Dominguez et al., 2021; Ye et al., 2023). Recent research has also emphasized the impact of genetic predispositions and social determinants on cognitive decline. Social factors such as isolation, lower socioeconomic status, and a lack of opportunities for cognitive engagement have been shown to exacerbate cognitive decline (Wang et al., 2023).

Focusing on modifiable lifestyle factors, such as smoking, alcohol consumption, and sedentary activities like watching television, is crucial for improving cognitive health in later life. These habits are prevalent and interconnected, but their precise impact on cognitive function remains unclear. Understanding their combined impact necessitates a comprehensive approach that considers how they interact and affect cognitive health collectively.

The MR approach offers significant advantages in elucidating the causal relationships between modifiable lifestyle habits and cognitive decline. By leveraging genetic variants as instrumental variables, MR helps to overcome confounding factors and reverse causality, which are common limitations in observational studies. This method enables more reliable identification of specific lifestyle factors. Our MVMR analyses further explored the causal associations among the three habits between smoking, alcohol intake, and television watching and cognitive function, revealing that time spent watching TV independently and predominantly accounted for these associations. After adjusting for BMI, education, and the number of days of moderate exercise/walking over 10 min, the effects were attenuated from OR 0.443 (95% CI: 0.341 to 0.574, *p* < 0.001) to 0.659 (95% CI: 0.452 to 0.960, *p* = 0.030; Figures 3, 4); however, the effects remained significant.

In our study, we excluded the confounding effect of physical exercise on the causal link between time spent watching television and cognitive decline. However, we do not deny the protective effect of exercise on cognition. Epidemiological data (Willey et al., 2016; Blondell et al., 2014) and animal research (Kim et al., 2022; Sun et al., 2019) suggest that physical activity may have a beneficial effect on cognitive function. Further investigation is needed to determine if physical activity can protect against cognitive decline resulting from low energy expenditure.

While all sedentary behaviors, such as watching TV, using a computer, driving, and reading, are classified as sedentary activities, they may differ in their behavioral patterns and effects on the brain. Watching TV is typically a more passive activity, whereas using a computer or driving may involve a higher cognitive load, such as typing, browsing, gaming, and interaction. This could have different impacts on cognitive function. Watching television as a sedentary behavior primarily involves minimal physical exertion and a low level of cognitive engagement. To further clarify the impact of low physical exertion on cognition and whether it can be reversed by increasing physical activity, we conducted animal experiments.

To explore the effects of sedentary behavior and physical exercise on cognition, we established a sedentary animal model using a PI cage. We used three classic behavioral experiments—the Morris water maze, the EPM, and the open field test—to evaluate the behavioral changes in mice from multiple dimensions, including spatial memory, anxiety, and exploratory behavior. The behavioral experiments revealed that mice with lower physical activity exhibited worse spatial memory, motor ability, and interest in new space exploration, while regular physical exercise improved spatial memory ability and increased the desire to explore new spaces.

Synaptophysin and PSD-95 were chosen as phenotypic indicators because they directly reflect the quantity, quality, and plasticity of synapses, which are essential to the neurobiological foundations for maintaining cognitive functions, particularly learning and memory. The hippocampus, a vital brain region deeply involved in learning and memory, is particularly vulnerable to oxidative stress damage induced by reactive oxygen species (ROS; Sies, 2015). These indicators enable us to roughly assess neuronal function and damage in the hippocampus. A Western blot analysis confirmed a significant increase in both synaptophysin and PSD-95 protein levels in the hippocampus of the Se + PE group compared with the Se group. Treadmill exercise appears to improve spatial learning ability and alleviate anxiety-like behaviors in sedentary mice, possibly through modulating ROS-synaptophysin pathways. The cognitive benefits of treadmill exercise have also been reported in other studies (Moon et al., 2016; Caldwell et al., 2020). Treadmill exercise has been demonstrated to enhance cognitive function by promoting synaptic plasticity and reducing oxidative stress (Moon et al., 2016; Caldwell et al., 2020). Our findings align with these studies, as we have observed significant increases in the levels of synaptophysin and PSD-95 levels in the Se + PE group compared to the Se group.

Our study employs large GWAS datasets and MVMR analysis to robustly explore the causal relationships between detrimental lifestyle habits and cognitive function. By incorporating a multivariable genetic model, we distinctly identify the separate effects of individual habits on cognitive health by effectively controlling for confounding and reverse causality. Furthermore, our behavioral and histological findings provide empirical evidence supporting the significant impact of sedentary behavior on cognitive decline.

However, our findings should be interpreted with caution, as there are several limitations to consider. First, although we use MVMR to explore the potential for horizontal pleiotropy due to observed confounding factors, MVMR cannot address biases arising from pleiotropic effects through other pathways than education, BMI, and exercise. Second, the generalizability of our results to broader populations should be carefully considered, as they may not be applicable to all demographic groups.

In conclusion, our analyses using UVMR and MVMR suggest that time spent watching television is a significant risk factor for cognitive decline. This activity, which is indicative of broader detrimental lifestyle habits such as smoking initiation and frequent alcohol consumption, appears to contribute independently and significantly to this association. Additionally, our animal study provides robust evidence that sedentary behavior negatively affects cognitive performance. These findings underscore the importance of targeted lifestyle interventions in reducing the risk of cognitive decline in older populations.

## Data Availability

The original contributions presented in the study are included in the article/Supplementary material, further inquiries can be directed to the corresponding authors.
