# Supplementary material for: Exploring the link between sedentary behavior and cognitive decline: a comprehensive study combining Mendelian randomization and animal model experiments
Source: Front Psychol. 2024 Oct 14;15:1407846. doi: 10.3389/fpsyg.2024.1407846 (PMC11513369; doi:10.3389/fpsyg.2024.1407846)
Supplement: Supplementary file 1 [file Data_Sheet_1.DOCX]

**
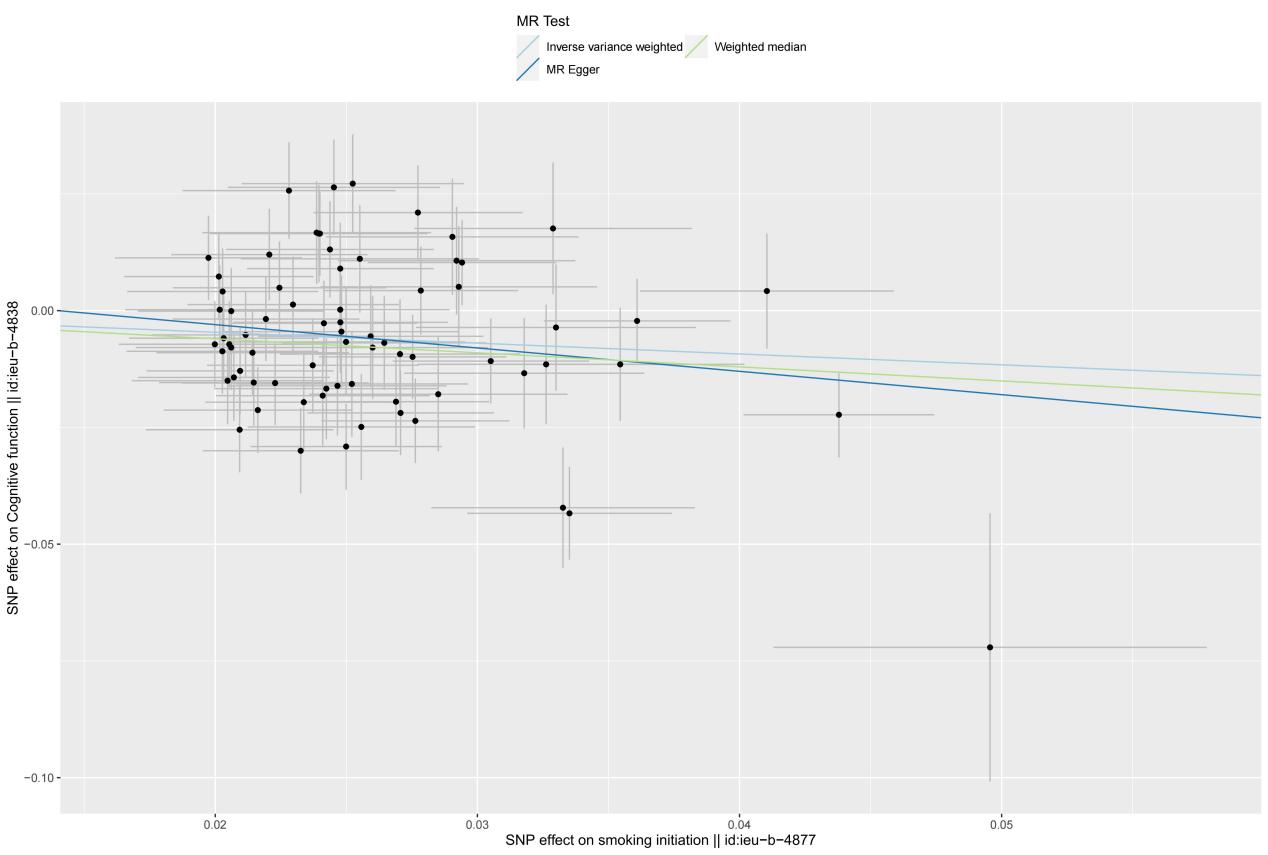
**

**Supplementary Figure 4. Scatter plot for UVMR analysis of causal relationship between** **"****smoking initiation" and worse cognitive function using three MR methods.**

The β value with SE is plotted to demonstrate effect estimate of each SNP (*n* = 73) for causal association of "**smoking initiation**" (x-axis) with **cognitive function**(y-axis). The slope of each line represents the UVMR estimate (β value) for the individual SNP. Error bar represents SE of effect size. **Abbreviations:** MR = Mendelian randomization; SE = standard error; SNP = number of single-nucleotide polymorphism; UVMR = univariate Mendelian randomization.


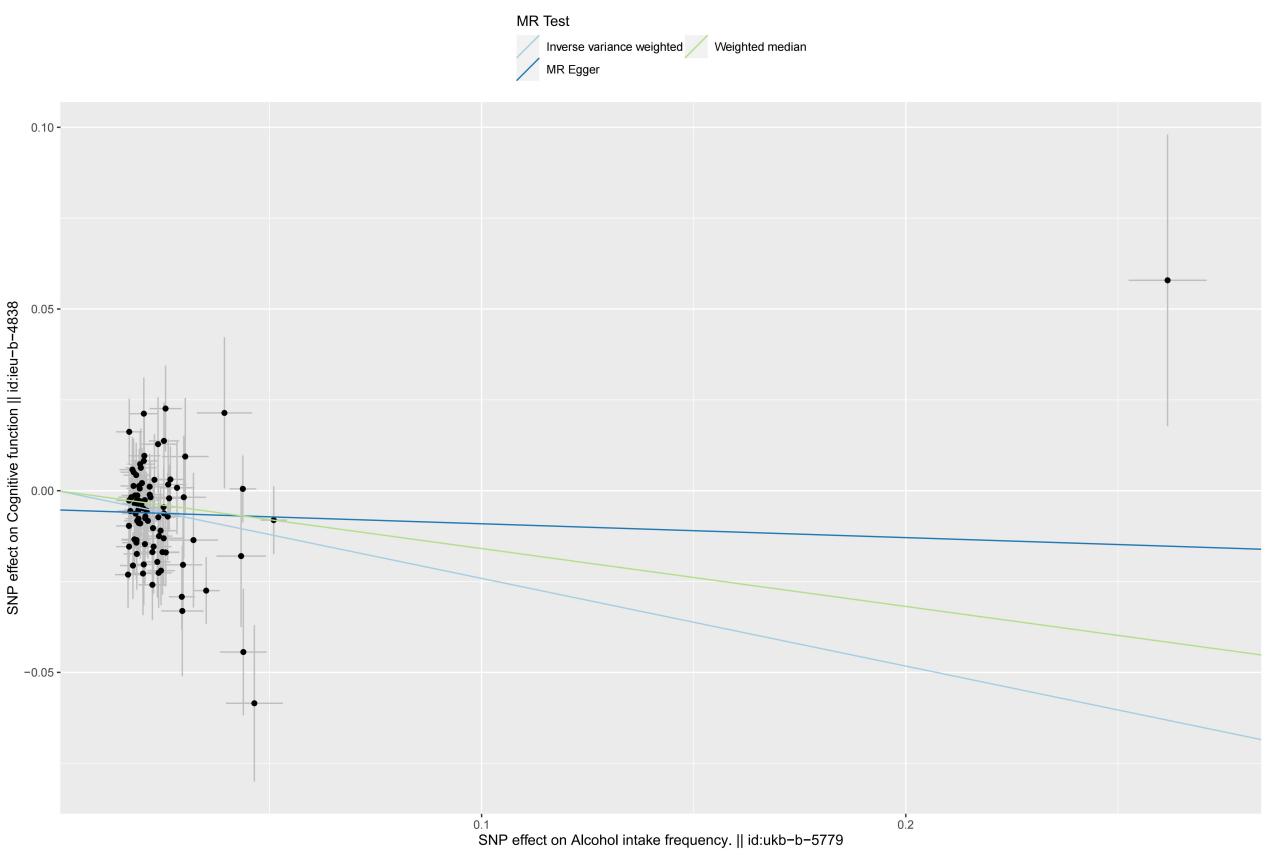


**Supplementary Figure 5. Scatter plot for UVMR analysis of causal relationship between "Alcohol intake frequency" and worse cognitive function using three MR methods.**

The β value with SE is plotted to demonstrate effect estimate of each SNP (*n* = 88) for causal association of "**Alcohol intake frequency**" (x-axis) with **cognitive function**(y-axis). The slope of each line represents the UVMR estimate (β value) for the individual SNP. Error bar represents SE of effect size. **Abbreviations:** MR = Mendelian randomization; SE = standard error; SNP = number of single-nucleotide polymorphism; UVMR = univariate Mendelian randomization.


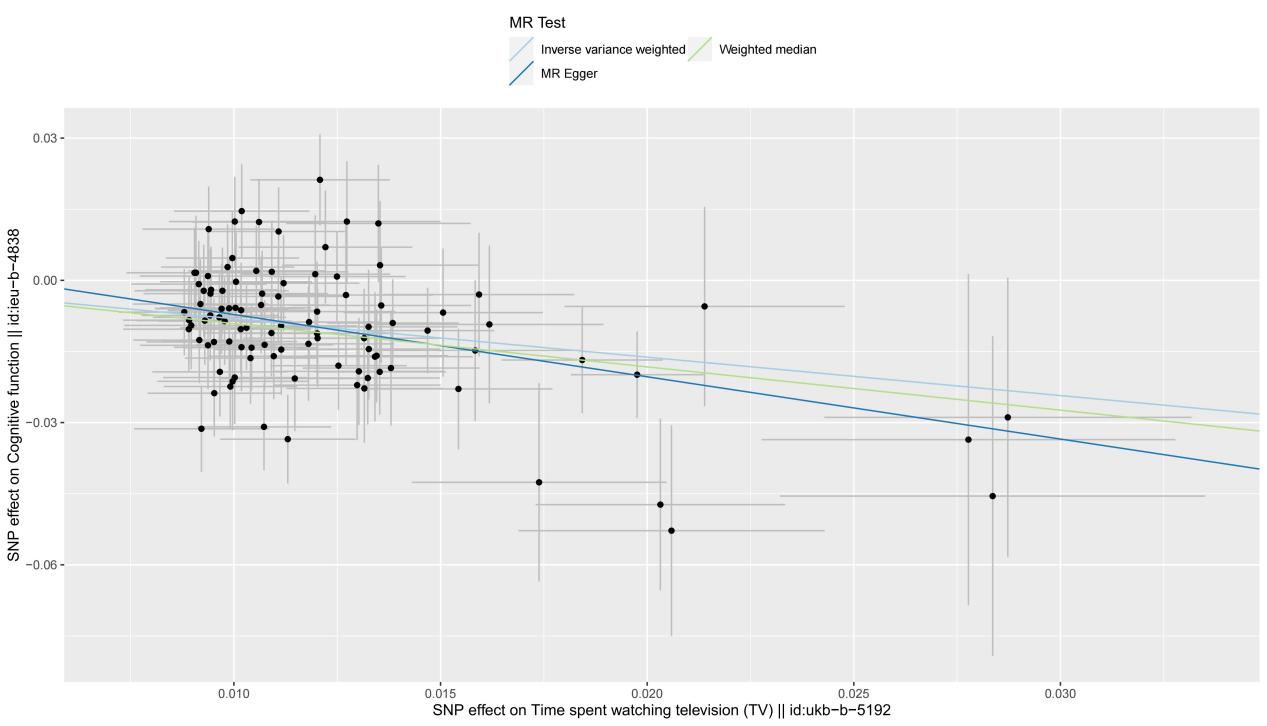


**Supplementary Figure 6. Scatter plot for UVMR analysis of causal relationship between "Time spent watching television" and worse cognitive function using three MR methods.**

The β value with SE is plotted to demonstrate effect estimate of each SNP (*n* = 100) for causal association of "**Time spent watching television**" (x-axis) with **cognitive function**(y-axis). The slope of each line represents the UVMR estimate (β value) for the individual SNP. Error bar represents SE of effect size. **Abbreviations:** MR = Mendelian randomization; SE = standard error; SNP = number of single-nucleotide polymorphism; UVMR = univariate Mendelian randomization.
