# Supplementary material for: Exploring the link between sedentary behavior and cognitive decline: a comprehensive study combining Mendelian randomization and animal model experiments
Source: Front Psychol. 2024 Oct 14;15:1407846. doi: 10.3389/fpsyg.2024.1407846 (PMC11513369; doi:10.3389/fpsyg.2024.1407846)
Supplement: Supplementary file 2 [file Data_Sheet_2.DOCX]

**
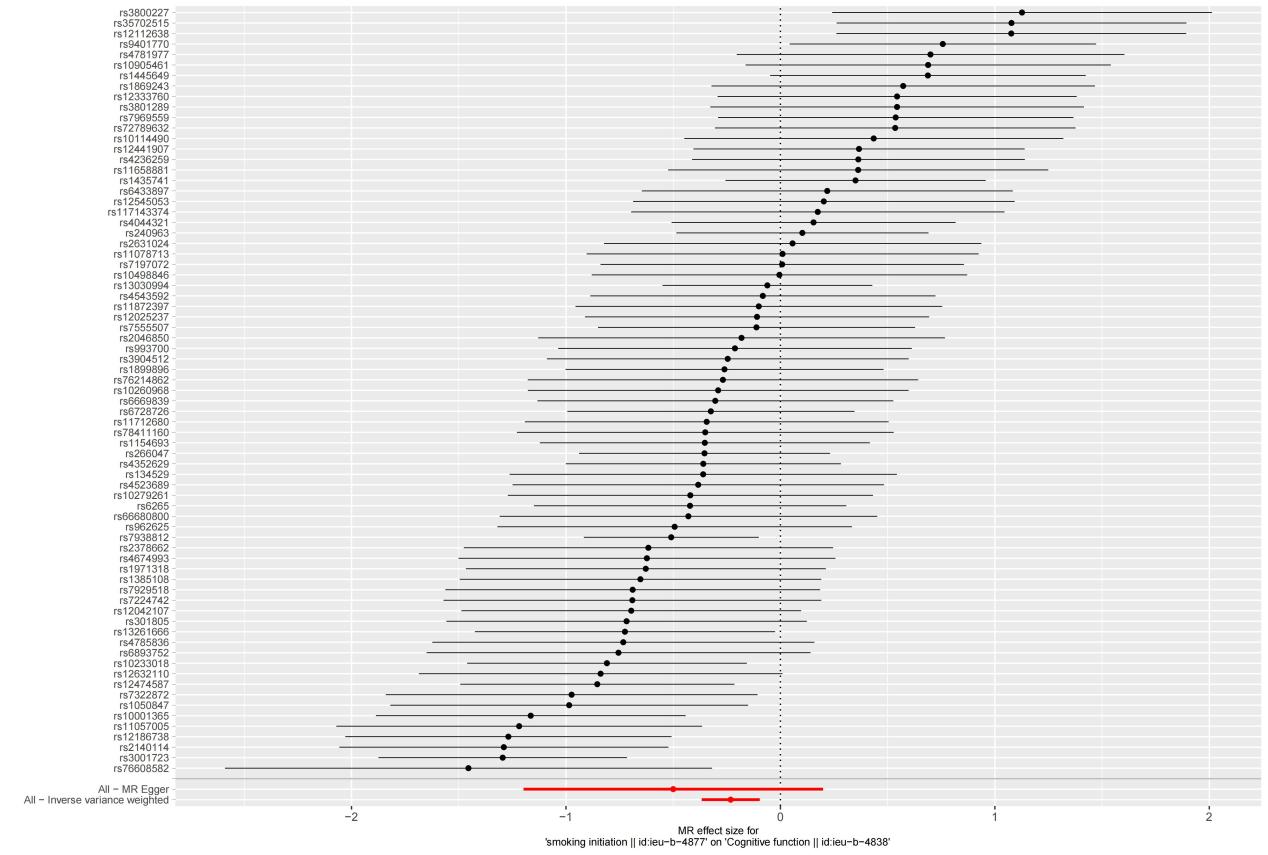
**

**Supplementary Figure 13. Forest plot for UVMR analysis of single and summarized SNPs effects on relationship between smoking initiation and** **cognitive function with 73 individual SNPs.**

A black point denotes the effect estimate of **smoking initiation** on **cognitive function** using a single SNP, and the black line signifies the 95% CI of the estimate. The red point symbolizes overall effect estimate of **smoking initiation**  on **cognitive function** with 73 SNPs using the Egger and IVW method, and the red line indicates the 95% CI of the estimate. **Abbreviations:** SNP = number of single-nucleotide polymorphism; UVMR = univariate Mendelian randomization; CI = confidence interval.


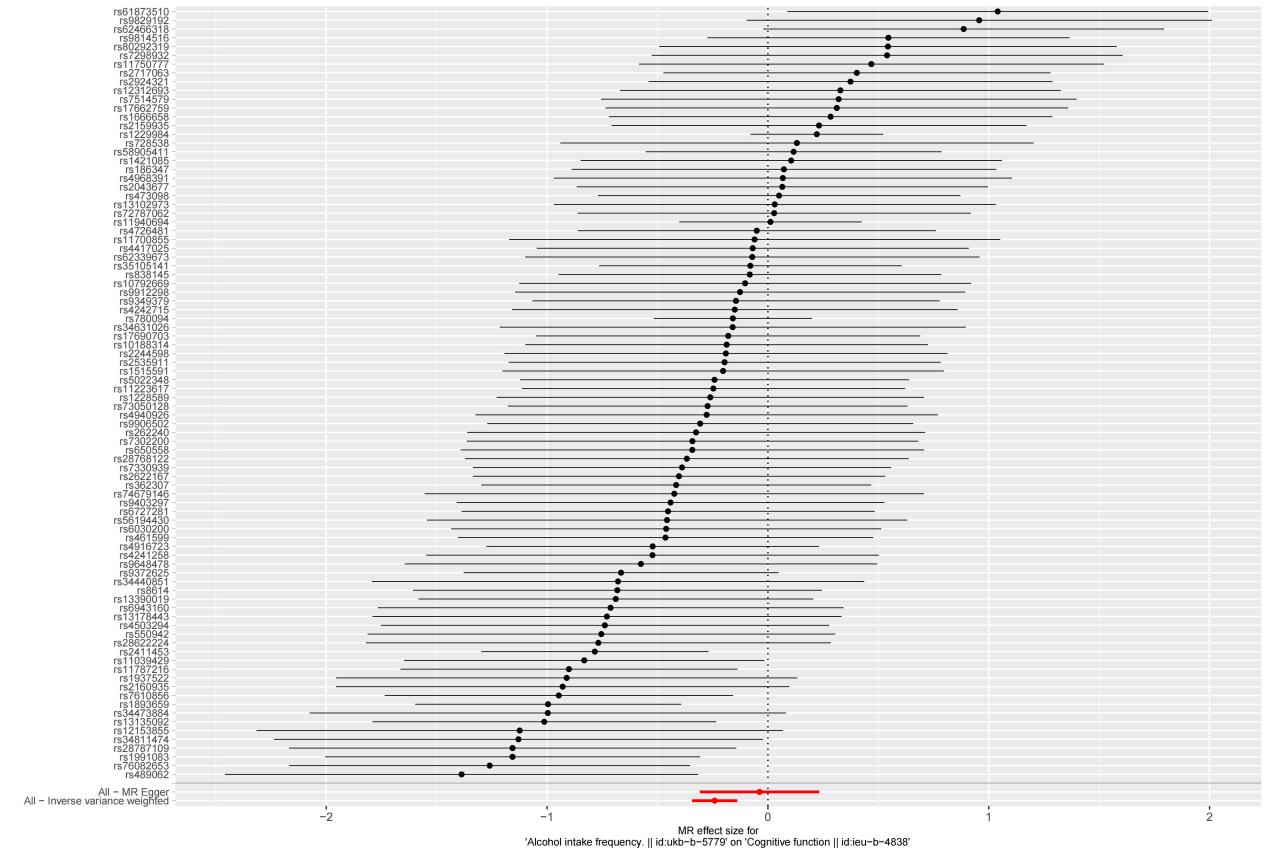


**Supplementary Figure 13. Forest plot for UVMR analysis of single and summarized SNPs effects on relationship between alcohol intake frequency and cognitive function with 88 individual SNPs.**

A black point denotes the effect estimate of **alcohol intake frequency** on **cognitive function** using a single SNP, and the black line signifies the 95% CI of the estimate. The red point symbolizes overall effect estimate of **alcohol intake frequency**  on **cognitive function** with 88 SNPs using the Egger and IVW method, and the red line indicates the 95% CI of the estimate. **Abbreviations:** SNP = number of single-nucleotide polymorphism; UVMR = univariate Mendelian randomization; CI = confidence interval.


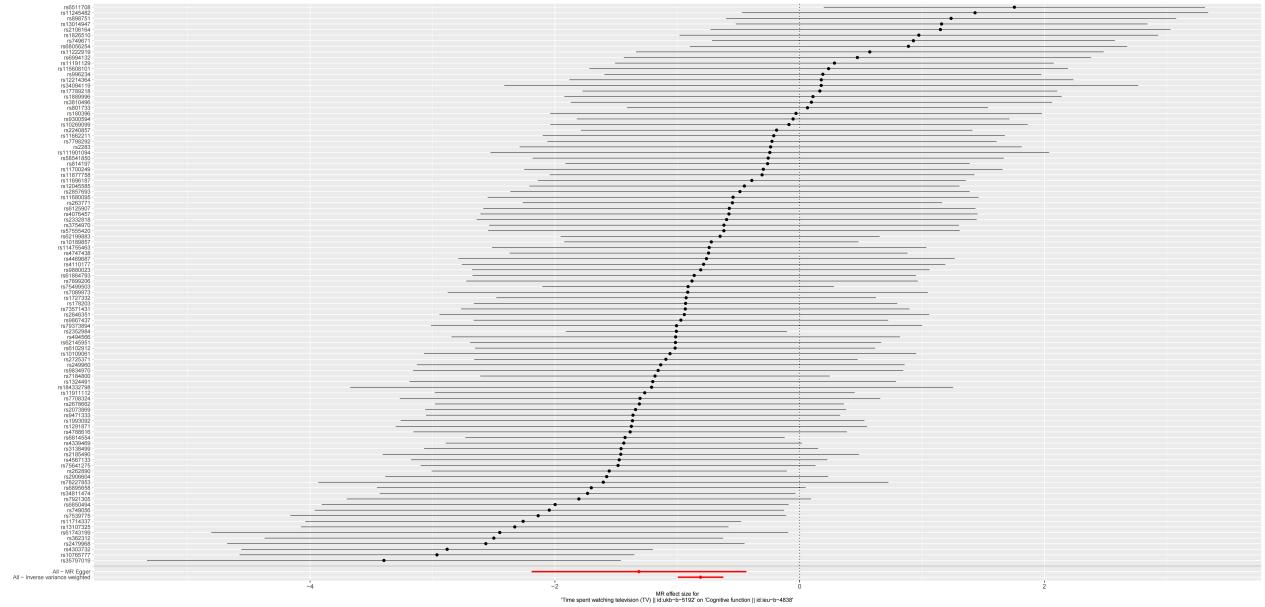


**Supplementary Figure 13. Forest plot for UVMR analysis of single and summarized SNPs effects on relationship between time spent watching television and cognitive function with 100 individual SNPs.**

A black point denotes the effect estimate of t**ime spent watching television** on **cognitive function** using a single SNP, and the black line signifies the 95% CI of the estimate. The red point symbolizes overall effect estimate of **time spent watching television**  on **cognitive function** with 88 SNPs using the Egger and IVW method, and the red line indicates the 95% CI of the estimate. **Abbreviations:** SNP = number of single-nucleotide polymorphism; UVMR = univariate Mendelian randomization; CI = confidence interval.
