# Supplementary material for: Exploring the link between sedentary behavior and cognitive decline: a comprehensive study combining Mendelian randomization and animal model experiments
Source: Front Psychol. 2024 Oct 14;15:1407846. doi: 10.3389/fpsyg.2024.1407846 (PMC11513369; doi:10.3389/fpsyg.2024.1407846)
Supplement: Supplementary file 3 [file Data_Sheet_3.DOCX]

**
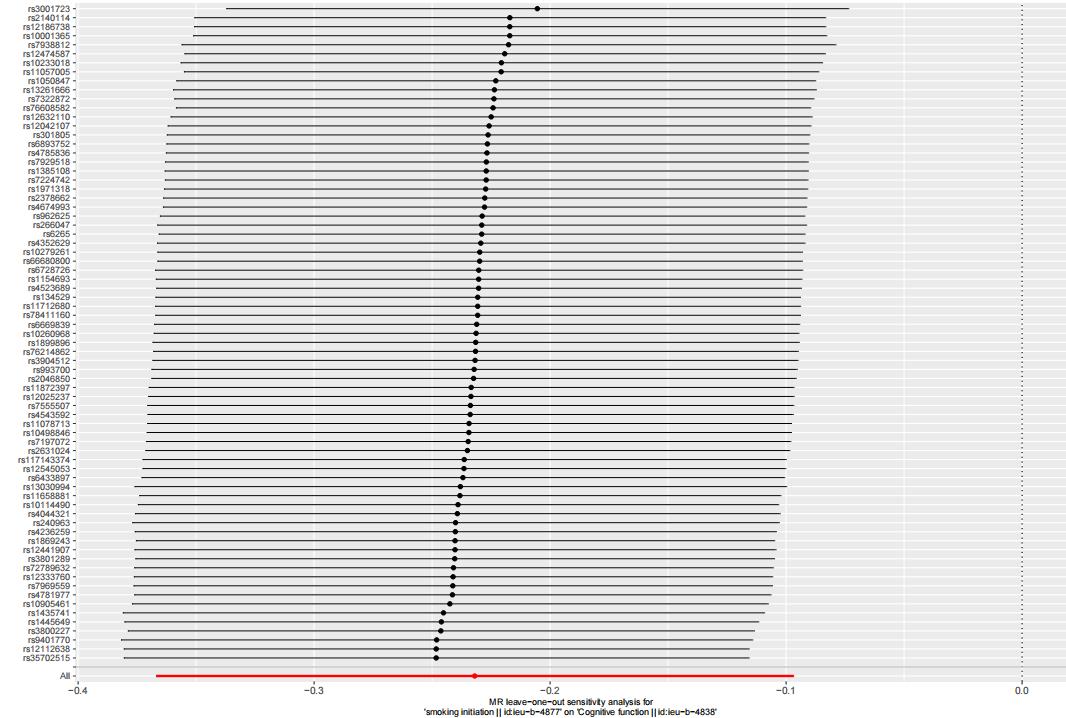
**

**Supplementary Figure 1. Leave-one-out plot for sensitivity analysis of single SNP effect on smoking initiation -to-****worse cognitive function UVMR results.**

Leave-one-out plot using IVW method by sequentially re-evaluating the causal estimate after discarding one SNP at a time, which helps determine whether the overall effect is driven by the specific genetic variant. The black point denotes the causal effect estimate of **smoking initiation** on **worse cognitive function** after discarding a certain SNP, and the black line signifies the 95% CI of estimate. The red point symbolizes the causal effect estimate of **smoking initiation** on **worse cognitive function** with the 73 valid SNPs, and the red line indicates the 95% CI of the estimate. **Abbreviations:** SNP = number of single-nucleotide polymorphism; UVMR = univariate Mendelian randomization; CI = confidence interval.


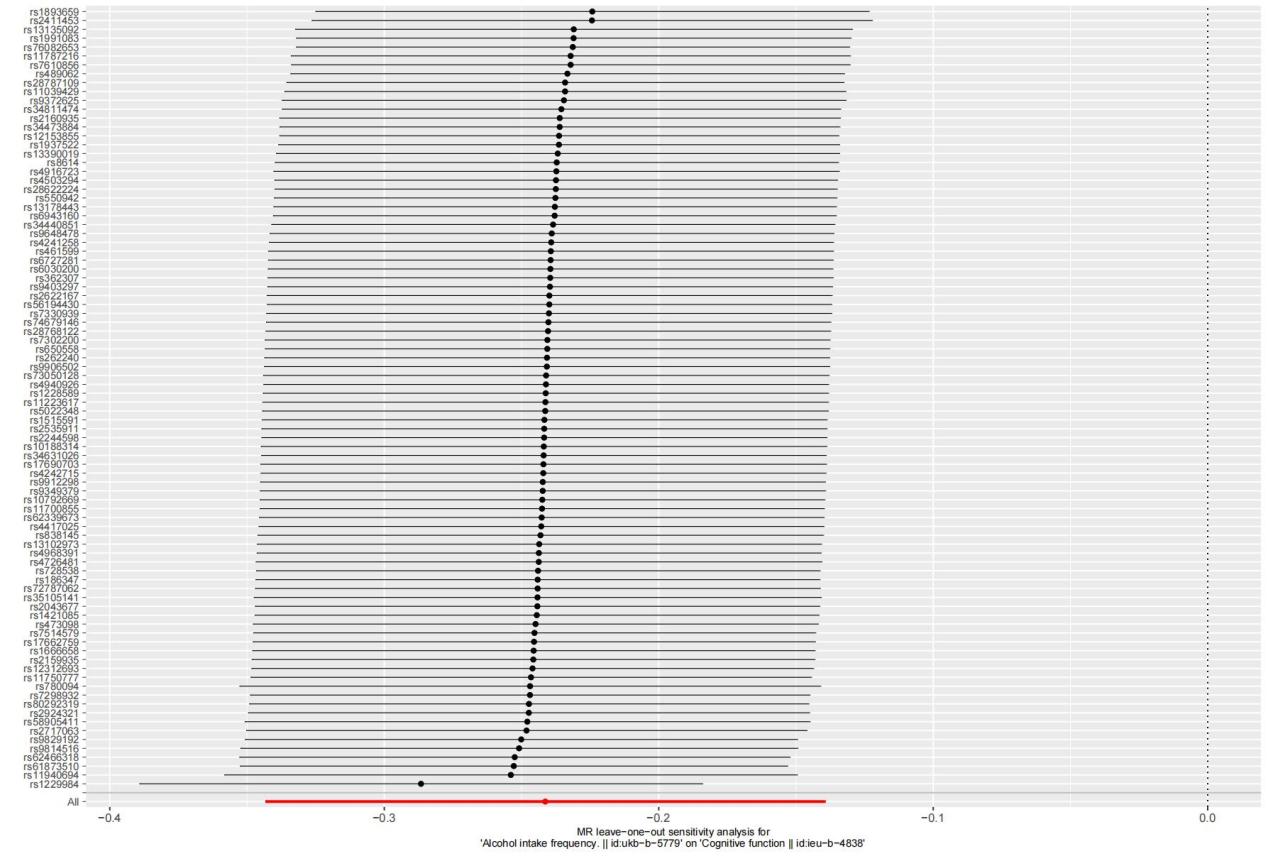


**Supplementary Figure 2. Leave-one-out plot for sensitivity analysis of single SNP effect on** **alcohol intake frequency -to-****worse cognitive function UVMR results.**

Leave-one-out plot using IVW method by sequentially re-evaluating the causal estimate after discarding one SNP at a time, which helps determine whether the overall effect is driven by the specific genetic variant. The black point denotes the causal effect estimate of **alcohol intake frequency** on **worse cognitive function** after discarding a certain SNP, and the black line signifies the 95% CI of estimate. The red point symbolizes the causal effect estimate of **alcohol intake frequency** on **worse cognitive function** with the 88 valid SNPs, and the red line indicates the 95% CI of the estimate. **Abbreviations:** SNP = number of single-nucleotide polymorphism; UVMR = univariate Mendelian randomization; CI = confidence interval.


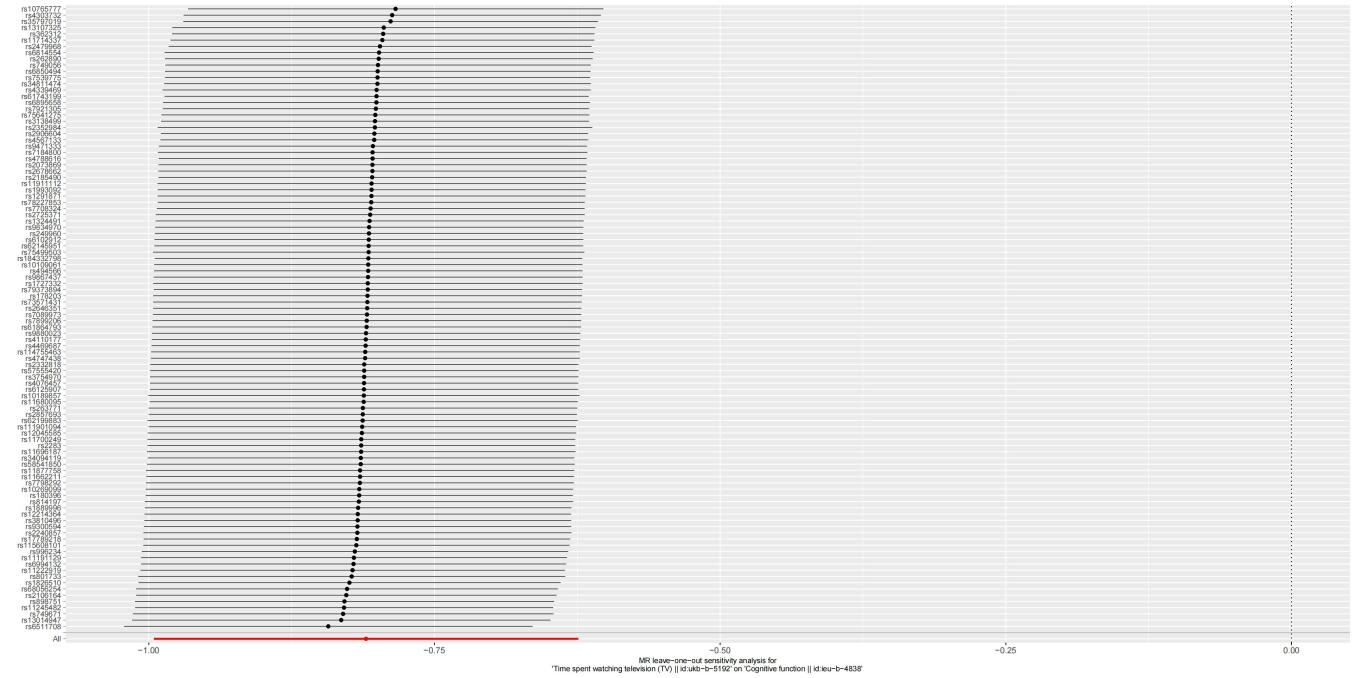


**Supplementary Figure 3. Leave-one-out plot for sensitivity analysis of single SNP effect on** **time spent watching television -to-worse cognitive function UVMR results.**

Leave-one-out plot using IVW method by sequentially re-evaluating the causal estimate after discarding one SNP at a time, which helps determine whether the overall effect is driven by the specific genetic variant. The black point denotes the causal effect estimate of **time spent watching television** on **worse cognitive function** after discarding a certain SNP, and the black line signifies the 95% CI of estimate. The red point symbolizes the causal effect estimate of **time spent watching television** on **worse cognitive function** with the 100 valid SNPs, and the red line indicates the 95% CI of the estimate. **Abbreviations:** SNP = number of single-nucleotide polymorphism; UVMR = univariate Mendelian randomization; CI = confidence interval.
